# The absolute value of recruitment-to-inflation ratio does not correlate with the recruited volume

**DOI:** 10.1186/s13054-023-04520-8

**Published:** 2023-06-21

**Authors:** Zhanqi Zhao, Mei-Yun Chang, Chan-Ching Chu, Hou-Tai Chang, Knut Möller, Inéz Frerichs, Yeong-Long Hsu

**Affiliations:** 1 Institute of Technical Medicine, Furtwangen University, Villingen-Schwenningen, Germany; 2 Department of Chest Medicine, Far Eastern Memorial Hospital, No. 21, Sec. 2, Nanya S. Rd., Banciao Dist., New Taipei City, Taiwan; 3 Department of Healthcare Management, College of Medical Technology and Nursing, Yuanpei University of Medical Technology, No. 306 Yuanpei Street, Hsinchu, Taiwan; 4 Department of Critical Care Medicine, Far Eastern Memorial Hospital, New Taipei City, Taiwan; 5 Department of Industrial Engineering and Management, Yuan Ze University, Taoyüan, Taiwan; 6 Department of Anaesthesiology and Intensive Care Medicine, University Medical Centre of Schleswig-Holstein Campus Kiel, Kiel, Germany; 7 Department of Electrical Engineering, Yuan Ze University, Taoyuan, Taiwan

The recruitment-to-inflation ratio (*R*/*I*) was proposed to assess recruitability in patients with acute respiratory distress syndrome (ARDS) [1]. The method calculates the compliance *C*_rec_ with the recruited volume and pressure differences between two positive end-expiratory pressures (PEEPs). *R*/*I* is the ratio between *C*_rec_ and the respiratory system compliance (*C*_rs_) at lower PEEP (PEEP_low_). In previous studies, it was demonstrated that overdistension could occur within tidal breathing, even when lung protective tidal volume was applied [2]. Therefore, the influence of overdistension should not be neglected for PEEP changes. Since the global compliance alone cannot distinguish atelectasis and overdistension, we hypothesized that *R*/*I* rather reflects a combination of recruitment and overdistension.

We evaluated the ARDS patients admitted to our center from 04.2017 to 06.2022 and participating in other studies (one was published NCT03112512). Sixty-two patients were screened and finally 58 patients analyzed (PaO_2_/FiO_2_ = 82.9 ± 30.0 mmHg). Four patients were excluded due to either no ventilator data or no electrical impedance tomography (EIT) data recorded. The patients were ventilated with lung protective ventilation strategies (low tidal volume ~ 6 ml/kg and individualized PEEP). PEEP was increased by 10 cmH_2_O (2 min PEEP_high_, 19.0 ± 2.5 cmH_2_O). Afterward, PEEP was decreased to the previous level (PEEP_low_, 9.3 ± 2.5 cmH_2_O). EIT measurement was conducted simultaneously with PulmoVista-500 (Draeger Medical, Germany) as specified by the device manufacturer. Relative impedance changes were calibrated to the corresponding volume changes in ml. Regional compliance was calculated for each pixel in the lung regions at both PEEP_high_ and PEEP_low_. Negative regional compliance change (Δ*C*_EIT_ = *C*_high_ − *C*_low_) indicated an overdistension at PEEP_high_. Positive value of Δ*C*_EIT_ suggested a recruitment at PEEP_high_. For calculation of *R*/*I*, the recruited volume was assessed with EIT as proposed in a previous study [3].

*C*_rs_ at PEEP_low_ was 39.5 ± 18.1 ml/cmH_2_O. *R*/*I* of the studied patients was 0.93 ± 0.69. The Δ*C*_EIT-overdistension_ was − 8.6 ± 7.3 ml/cmH_2_O and Δ*C*_EIT-recruitment_ was 6.1 ± 3.7 ml/cmH_2_O. The correlation between *R*/*I* and Δ*C*_EIT-recruitment_ was statistically insignificant (r = − 0.25). On the other hand, *R*/*I* and |Δ*C*_EIT-overdistension_|/Δ*C*_EIT-recruitment_ were significantly correlated (*r* = 0.31, *p* = 0.02).

Our study showed that *R*/*I* might not be a reliable index to assess recruitment but rather has a weak correlation with the mixture of recruitment and overdistension. The calculation of *R*/*I* holds several assumptions (e.g., linear *C*_rec_ within ΔPEEP and *C*_rs_ within tidal breathing at PEEP_low_). Only when these assumptions are met, *R*/*I* reflects solely the recruitability (e.g., overdistension is not present at either PEEP_low_ or PEEP_high_). Volume-dependent compliance changes have been intensively studied, and the results suggested that intra-tidal *C*_rs_ is not necessarily linear in ARDS. Using *C*_rs_ value at PEEP_low_ to predict the volume change in already aerated lung regions could be misleading (Fig. 1). Besides, *R*/*I* neglects the fact that intra-tidal overdistension may occur at PEEP_high_ [2]. *R*/*I* was correlated with PaO_2_/FiO_2_ and dead space in the original study [1], but those measures did not provide a direct proof of recruitability. Regional EIT information is used at the bedside to identify recruitment and overdistension [2, 3]. Therefore, we utilized the data set to test our hypothesis. A recent study obtained opposite results to ours [4]. We speculated that 1. PEEP_high_ value selected in that study was the optimal PEEP decided by EIT, at which little overdistension might have been present, and 2. the overdistension and recruitment calculated in that study were relative to the maximum regional compliance [3]. The resulting values depend on the starting and ending PEEP levels of the PEEP titration, as well as the number of PEEP steps. Due to the calculation limitation of the relative compliance change, the percentage of overdistension at the lowest PEEP would be 0 regardless of the reality. On the other hand, we calculated the absolute changes of regional compliance, which would not have the limitations discussed above and more accurately reflect the degree of overdistension and recruitment. In another study [5], Taenaka et al*.* found weak correlation between *R*/*I* and *C*_rs_, *R*/*I* and silent spaces (presumably lung collapse and overdistension), which coincided to our findings that *R*/*I* assessed not only recruitment but also overdistension.

**Fig. 1:**
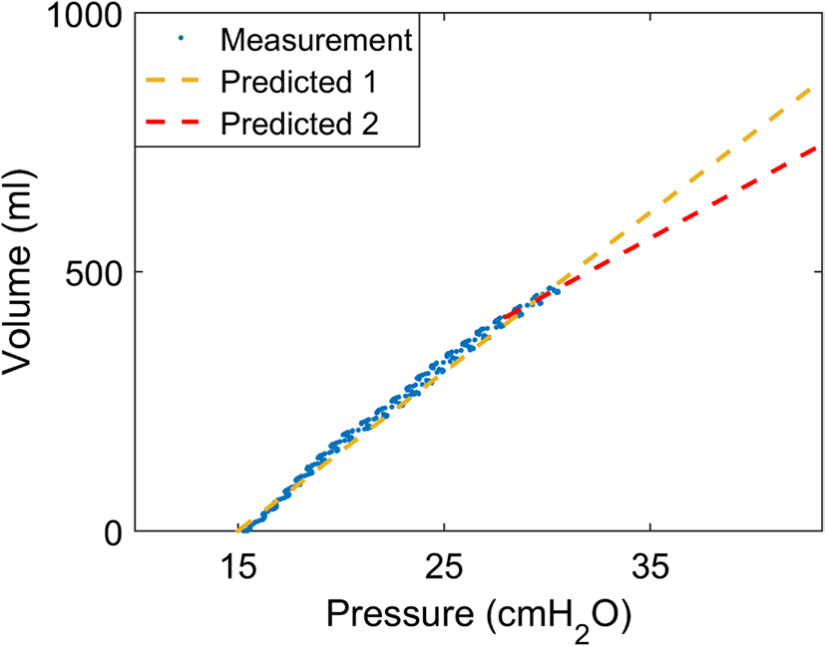
Pressure–volume curve from a study subject with overdistension at PEEP_high_. Predicted volume change using the global respiratory compliance (Predicted 1) and using the volume-dependent compliance toward the end of inspiration (Predicted 2)

As limitation, the present study was a retrospective analysis of prospective studies. The calculation of *R*/*I* was not according to the original publication [1] but rather an alternative [4], which is also widely used. In the original publication, lowest pressure for opening the airways should be identified, which was not assessed in the current study. We could not rule out the possibility that PEEP_low_ might have been lower than the airway opening pressure in some patients. On the other hand, PEEP_low_ applied in the current study was considered an adequate PEEP level for the patients; therefore, PEEP_high_ might have introduced considerable overdistension compared to the original study data. Furthermore, the compliance increase might not be linearly related to the recruited volume.

Nevertheless, *R*/*I* may ignore the overdistension and could be misleading if the absolute value is used to guide ventilator settings alone.

## Data Availability

Data are available upon reasonable request from the corresponding author.
